# Proteomics and Phosphoproteomics of Heat Stress-Responsive Mechanisms in Spinach

**DOI:** 10.3389/fpls.2018.00800

**Published:** 2018-06-26

**Authors:** Qi Zhao, Wenxin Chen, Jiayi Bian, Hao Xie, Ying Li, Chenxi Xu, Jun Ma, Siyi Guo, Jiaying Chen, Xiaofeng Cai, Xiaoli Wang, Quanhua Wang, Yimin She, Sixue Chen, Zhiqiang Zhou, Shaojun Dai

**Affiliations:** ^1^Development Center of Plant Germplasm Resources, College of Life and Environmental Sciences, Shanghai Normal University, Shanghai, China; ^2^Key Laboratory of Forest Plant Ecology, Key Laboratory of Saline-alkali Vegetation Ecology Restoration, Ministry of Education, Alkali Soil Natural Environmental Science Center, Northeast Forestry University, Harbin, China; ^3^Institute of Life Sciences, Chongqing Medical University, Chongqing, China; ^4^Shanghai Center for Plant Stress Biology, Chinese Academy of Sciences, Shanghai, China; ^5^Institute of Plant Stress Biology, State Key Laboratory of Cotton Biology, Department of Biology, Henan University, Kaifeng, China; ^6^Plant Molecular and Cellular Biology Program, Department of Biology, Genetics Institute, Interdisciplinary Center for Biotechnology Research, University of Florida, Gainesville, FL, United States

**Keywords:** heat adaptation, signal transduction, ROS homeostasis, spinach, proteomics

## Abstract

Elevated temperatures limit plant growth and reproduction and pose a growing threat to agriculture. Plant heat stress response is highly conserved and fine-tuned in multiple pathways. Spinach (*Spinacia oleracea* L.) is a cold tolerant but heat sensitive green leafy vegetable. In this study, heat adaptation mechanisms in a spinach sibling inbred heat-tolerant line Sp75 were investigated using physiological, proteomic, and phosphoproteomic approaches. The abundance patterns of 911 heat stress-responsive proteins, and phosphorylation level changes of 45 phosphoproteins indicated heat-induced calcium-mediated signaling, ROS homeostasis, endomembrane trafficking, and cross-membrane transport pathways, as well as more than 15 transcription regulation factors. Although photosynthesis was inhibited, diverse primary and secondary metabolic pathways were employed for defense against heat stress, such as glycolysis, pentose phosphate pathway, amino acid metabolism, fatty acid metabolism, nucleotide metabolism, vitamin metabolism, and isoprenoid biosynthesis. These data constitute a heat stress-responsive metabolic atlas in spinach, which will springboard further investigations into the sophisticated molecular mechanisms of plant heat adaptation and inform spinach molecular breeding initiatives.

## Introduction

Heat stress has become a major deleterious factor affecting crop production worldwide due to global climate change (Wahid et al., 2007). Heat stress directly leads to protein denaturation, aggregation, and increases fluidity of membrane lipids, but also indirectly results in inactivation of enzymes in chloroplasts and mitochondria, inhibition of protein synthesis and degradation, as well as impairment of membrane integrity (Wahid et al., 2007; Bokszczanin et al., 2013). Therefore, a better understanding of plant thermotolerant mechanisms is essential toward breeding heat-tolerant crop plants.

To counteract the detrimental effects of heat stress, plants have employed multiple stress-tolerant strategies by altering the gene expression, protein synthesis, and post-translational modification, which contribute to the reestablishment of cellular homeostasis for plant survival under high temperature (Bokszczanin et al., 2013). Recent transcriptomic studies have identified a large number of heat stress-responsive genes closely related with heat tolerance in various plant species including *Arabidopsis thaliana* (Higashi et al., 2015), rice (*Oryza sativa*) (Zhang et al., 2013), maize (*Zea mays*) (Li et al., 2017), spinach (*Spinacia oleracea*) (Yan et al., 2016), potato (*Solanum tuberosum*) (Hancock et al., 2014), grape (*Vitis vinifera*) (Rocheta et al., 2014; Jiang et al., 2017), perennial ryegrass (*Lolium perenne*) (Wang K. et al., 2017), *Rhazya stricta* (Obaid et al., 2016), carnation (*Dianthus caryophyllus*) (Wan et al., 2015), and *Saccharina japonica* (Liu F. et al., 2014). These heat stress-responsive genes were involved in various biological processes such as transcription regulation, alternative splicing, antioxidant defense, photosynthesis, protein homeostasis, as well as metabolisms of carbohydrates, lipids, amino acids, and secondary metabolites. Among them, most genes encoding transcription factors and heat shock proteins (HSPs) were up-regulated. These changes are necessary for regulating gene expression and maintaining protein stability under heat stress.

It is well-established that a poor correlation between transcript and protein levels exists because of post-transcriptional and post-translational modifications. Proteomics offers a powerful approach to discover heat stress-responsive proteins (HRPs) and elucidate pathways that are crucial for heat tolerance (Wang X. et al., 2017). Proteomic responses to heat stress have been investigated in leaves from different plants, including Arabidopsis (Rocco et al., 2013), alfalfa (*Medicago sativa*) (Li et al., 2013), rice (Lee et al., 2007; Han et al., 2009; Das et al., 2015), wild rice (*Oryza meridionalis*) (Scafaro et al., 2010), wheat (*Triticum aestivum*) (Wang et al., 2015; Lu et al., 2017), wild barley (*Hordeum spontaneum*) (Rollins et al., 2013; Ashoub et al., 2015), maize (Hu et al., 2015; Zhao F. et al., 2016), soybean (*Glycine max*) (Ahsan et al., 2010; Das et al., 2016), celery (*Apium graveolens*) (Huang et al., 2017), grape (Liu G.T. et al., 2014; Jiang et al., 2017), *Pinellia ternata* (Zhu et al., 2013), *Carissa spinarum* (Zhang et al., 2010), *Portulaca oleracea* (Yang et al., 2012), *Agrostis scabra* and *A. stolonifera* (Xu and Huang, 2010), *Populus euphratica* (Ferreira et al., 2006), and *Populus yunnanensis* (Li et al., 2014). Numerous proteins related to signal transduction, photosynthesis, antioxidant defense, transcriptional and post-transcriptional regulation, protein synthesis and turnover, carbohydrate and energy metabolism were found to play pivotal roles in protecting leaves against heat stress. Importantly, protein phosphorylation is required for regulating a wide range of cellular processes and signal relays in response to heat stress. A phosphoproteomics study has reported that maize leaf phosphoproteins play critical roles in signal transduction cascades, transport of water, sugar and H^+^, gluconeogenesis, and protein homeostasis during heat response (Hu et al., 2015). These results have enriched our knowledge of molecular mechanisms underlying plant tolerance to heat stress.

Spinach, a nutritious green leafy vegetable, is cold tolerant but heat sensitive. Its growth and development are affected by heat stress, leading to significant decreases in yield and quality. Therefore, unraveling the heat stress-responsive mechanisms in spinach is of great importance toward molecular breeding of heat-tolerant cultivars. Previous studies on the response of spinach to heat stress have mainly focused on physiological changes. For instance, photosynthesis in spinach was shown to be highly sensitive to heat stress (Thebud and Santarius, 1982). Photosystem II (PSII) activity was inhibited because of the heat-induced aggregation of light-harvesting complex II, cleavage and aggregation of the D1 protein, and release of the extrinsic proteins (PsbO, PsbP, and PsbQ) (Yoshioka et al., 2006; Komayama et al., 2007; Tang et al., 2007; Yamashita et al., 2008). In addition, ribulose bisphosphate carboxylase/oxygenase (RuBisCO) activase (RCA) was considered to limit photosynthesis under heat stress (Salvucci and Crafts-Brandner, 2004a). Moreover, it was reported that heat-increased thermostability of the leaves might be attributed to the structural stabilization of PSII submembrane fraction, thylakoid membrane, and chloroplast envelope (Santarius, 1973; Krause and Santarius, 1975; Santarius and Müller, 1979; Allakhverdiev et al., 1996). Recently, a transcriptomic analysis of spinach leaves led to the identification of 896 unique genes in spinach heat stress response, and they play important roles in signal transduction, reactive oxygen species (ROS) homeostasis, transcription regulation, and protein stability under heat stress (Yan et al., 2016). However, only a few heat stress-responsive genes and proteins in spinach have been characterized, such as glycinebetaine biosynthesis-related genes, *choline monooxygenase*, and *betaine aldehyde dehydrogenase*. The plants overexpressing spinach *choline monooxygenase* and *betaine aldehyde dehydrogenase* genes enhanced heat tolerance (Yang et al., 2005; Shirasawa et al., 2006).

In spite of this information, global proteomic studies are lacking in spinach. Here we conducted proteomics and phosphoproteomics combined with physiological analyses in leaves from a heat-tolerant spinach sibling inbred line Sp75. A total of 911 proteins and 45 phosphoproteins were identified in response to the heat stress. These results have improved our understanding of spinach heat adaptation mechanisms, and provided new perspectives for improving spinach thermotolerance.

## Materials and Methods

### Plant Material and Heat Treatments

A sibling inbred line of spinach (*Spinacia oleracea* L.), Sp75, was grown in a growth chamber with a temperature regime of 22/18°C, 10/14 h day/night cycle, and a relative humidity of 60%. Plants were watered daily to avoid the occurrence of water deficit. When performing heat treatments, plants of the treatment groups were moved to another growth chamber with the same growth condition as the control, except for temperature (37/32°C day/night), and watered daily on a regular schedule as well. The 72 h of heat treatment was started at 10:00 a.m. on the 50th day after seeds were sown, and 48 and 24 h of heat treatments were conducted at 10:00 a.m. on the 51th day and 52th day, respectively. After heat treatments, fully expanded true leaves were collected, morphological changes were recorded, and photosynthetic characteristics were measured for both control and heat-treated plants at 10:00 a.m. on the 53rd day to avoid the differences caused by plant growth, development, and circadian rhythms (Supplementary Figure S1). Samples were either used fresh or immediately frozen in liquid nitrogen and stored at -80°C.

### Relative Water Content (RWC) Measurement

For determination of RWC, 0.2 g fresh leaves were detached, weighed immediately, and recorded as fresh weight. Then the leaves were soaked in distilled water for 24 h, the turgid weight was quickly measured, and the dry weight was recorded after drying at 80°C for 2 h followed by 60°C to a constant weight. The RWC was calculated as: RWC = [(Fresh weight-Dry weight)/(Turgid weight-Dry weight)] × 100%.

### Photosynthesis and Chlorophyll Fluorescence Analyses

Net photosynthetic rate (Pn), stomatal conductance (Gs), intercellular CO_2_ concentration (Ci), and transpiration rate (Tr) were determined at 10:00 a.m. using a portable photosynthesis system LICOR 6400 (LI-COR Inc., Lincoln, NE, United States). The chlorophyll fluorescence parameters were recorded by using a portable modulated chlorophyll fluorometer (Model OS5p+, OPTI-Sciences, Hudson, NH, United States).

### Proline, Total Soluble Sugar, Malondialdehyde (MDA) and Relative Electrolyte Leakage (REL) Measurements

Proline and total soluble sugar contents were determined using ninhydrin reaction and a sulfuric acid-anthrone reagent according to previous methods, respectively (Li et al., 2000). MDA content and REL were measured using previous methods (Wang et al., 2010).

### H_2_O_2_ Content, Antioxidant Enzyme Activity, and Antioxidant Content Assays

For H_2_O_2_ measurement, leaf tissue was ground with 0.1% trichloroacetic acid. After centrifugation at 15,000 *g* for 15 min at 4°C, the supernatant was collected for H_2_O_2_ measurement. H_2_O_2_ content was determined spectrophotometrically at 390 nm after reacting with potassium iodide as described in a previous study (Ibrahim and Jaafar, 2012). The activities of superoxide dismutase (SOD), peroxidase (POD), glycolate oxidase (GOX), catalase (CAT), ascorbate peroxidase (APX), monodehydroascorbate reductase (MDHAR), dehydroascorbate reductase (DHAR), glutathione reductase (GR), glutathione S-transferase (GST), and glutathione peroxidase (GPX) were measured according to our previous methods (Yu et al., 2011; Zhao Q. et al., 2016). The contents of ascorbate (AsA), dehydroascorbate (DHA), glutathione (GSH), and oxidized glutathione (GSSG) were measured following previous methods (Yin et al., 2017).

### Protein Sample Preparation for Proteomics

Total leaf protein was extracted from four biological replicate samples for each treatment using a phenol extraction protocol (Wang et al., 2010). Protein pellets were dissolved in a lysis buffer (7 M urea, 2 M thiourea, 4% CHAPS, 0.04 M DTT, 4% proteinase inhibitor cocktail). The protein concentration was determined using a 2D Quant Kit (GE Healthcare, Uppsala, Sweden) according to the manufacturer’s instructions.

### Trypsin Digestion and iTRAQ Labeling

Protein reduction, alkylation, and digestion were performed via the filter-aided sample preparation (FASP) workflow (Abdallah et al., 2012; McDowell et al., 2013) by using a commercially available ultra-filtration device with a molecular weight cutoff of 10 kDa (Sartorius Stedim Biotech, Göttingen, Germany) used for protein retention, buffer exchange, and removal. For each sample, 100 μg protein was mixed with 100 μl 0.05 M NH_4_HCO_3_ in the filter device, followed by centrifugation at 13,000 rpm at 18°C for 15 min. The protein sample was reduced using 50 mM DTT in 7 M guanidine-HCl (pH 8.0) at 45°C for 1 h, and then alkylated using 125 mM iodoacetamide in 7 M guanidine-HCl (pH 8.0) for 30 min in the dark at room temperature followed by centrifugation. The resulting concentrate was washed three times with 0.5 M triethylammonium bicarbonate at pH 8.5 to remove interfering compounds including DTT, iodoacetamide, and NH_4_HCO_3_. Trypsin was added to the samples at 1:50 (w/w, trypsin: sample) and incubated at 37°C for 16 h. The tryptic peptides in the filter device were collected by centrifugation and labeled with iTRAQ 8-plex reagents according to manufacturer’s instructions (AB Sciex, Framingham, MA, United States). The samples of 0 hour of heat treatment (HHT) were labeled with iTRAQ tags 113 and 117, 24 HHT with tags 114 and 118, 48 HHT with tags 115 and 119, and 72 HHT with tags 116 and 121. Two independent iTRAQ experiments were carried out, thus each sample has four biological replicates (Zhu et al., 2012; He et al., 2013). After labeling, the samples were combined, divided into two aliquots and lyophilized. Peptides from one aliquot were fractioned and desalted before it was used for HRP analysis, and peptides from the other aliquot were used for heat stress-responsive phosphoprotein analysis.

### Protein Fractionation and Desalination

The peptides were dissolved in solvent A (0.02 M ammonium formate, pH 10.5) and fractionated on a Shimadzu LC-20A HPLC system (Shimadzu, Kyoto, Japan) with a XBridge C18 column (150 mm × 4.6 mm, 5 μm, Waters, Milford, MA, United States). Peptides were eluted at a flow rate of 800 μl/min with a linear gradient of 5–55% solvent B (0.02 M ammonium formate in 80% acetonitrile, pH 10.5) over 42 min followed by ramping up to 90% solvent B in 1 min and holding for 4 min. The fractions were lyophilized and desalted using 3M Empore C18 solid-phase extraction disks (3M Bioanalytical Technologies, St. Paul, MN, United States).

### Enrichment of Phosphorylated Peptides Using TiO_2_ Beads

The labeled peptides were resuspended in 500 μl loading buffer (1% glutamic acid/65% acetonitrile/2% trifluoroacetic acid). TiO_2_ beads were added and the mixture was incubated for 30 min at room temperature. After centrifugation, the mixture was washed twice using wash buffer I (65% acetonitrile/0.5% trifluoroacetic acid) and wash buffer II (65% acetonitrile/ 0.1% trifluoroacetic acid), respectively. The phosphopeptides were then eluted with elution buffer I (0.3 M NH_4_OH/50% acetonitrile) and then elution buffer II (0.5 M NH_4_OH/60% acetonitrile). The eluted peptides were lyophilized and stored in -20°C.

### LC-MS/MS and Data Analysis

Samples were reconstituted in 10 μl of 0.1% formic acid and analyzed by online nanoAcquity ultraperformance LC (Waters, Milford, MA, United States) coupled with an Orbitrap Fusion Tribrid mass spectrometer (Thermo Fisher Scientific, San Jose, CA, United States) according to a previously described method (Ma et al., 2016).

MS/MS spectra were searched against a protein database (27,376 sequences^1^) (Xu et al., 2015, 2017) using Proteome Discoverer 2.1 software (Thermo Fisher Scientific, Bremen, Germany). The Sequest HT algorithm was used for protein identification. Trypsin was selected as the enzyme with two maximum missing cleavages. Mass error of precursor ions and fragment ions was set to 10 ppm and 0.02 Da, respectively. Carbamidomethyl (C), iTRAQ 8-plex (N-term), and iTRAQ 8-plex (K) were set as static modifications, and oxidation on methionine was specified as dynamic modifications. For phosphopeptide identification, a dynamic modification of phosphorylation(S/T/Y) was added to the above criteria. A strict and relaxed target false discovery rate was set as 0.01 and 0.05, respectively. Quantification was performed using iTRAQ reporter ion peak intensities. Proteins considered as reliably identified should meet the following criteria: number of unique peptides ≥ 2, number of razor peptides ≤ 1, and proteins false discovery rate ≤ 1%. The criteria for reliable phosphopeptides include: confidence = high, ambiguity = unambiguous, unique quantification, containing phosphorylation modification, number of protein group = 1, rank A Sequest HT = 1, and delta Cn A Sequest HT = 0. The ratio between samples was calculated using reporter ion intensity and normalized to the sum ratio of all identified spectra that fit above criteria. To designate the HRPs and heat stress-responsive phosphopeptides, a Student’s *t*-test *p* < 0.05 and fold change > 1.2 across the treatment and control samples in four replicates were applied. Moreover, a heat stress-responsive phosphopeptide should also meet another criterion that the fold change of peptide phosphorylation level should be higher than the corresponding protein abundance. The mass spectrometry proteomics data had been deposited to the ProteomeXchange Consortium (Vizcaino et al., 2014) via the PRIDE partner repository with the dataset identifier PXD009352.

### Protein Classification and Hierarchical Clustering

Proteins without detailed annotation were annotated by searching against the NCBI non-redundant protein database^2^ using PSI and PHI-BLAST programs^3^. Protein functional classification was performed manually on the basis of combination of information from KEGG pathway database^4^, UniProt database^5^, and the Gene Ontology protein database^6^, as well as literature. Hierarchical clustering analysis of the protein abundances in leaves was performed on the log (base 2) transformed protein fold change values using Cluster 3.0 available on the Internet^7^.

### Phosphoprotein Homology Modeling

Three-dimensional structural models for phosphoproteins were generated using SWISS-MODEL comparative protein modeling server^8^ (Biasini et al., 2014). Structures were visualized and analyzed using the Swiss-PdbViewer software (version 3.7). Functional domains were predicted by InterPro: the integrative protein signature database^9^.

### Statistical Analysis

All results were presented as means ± standard deviation. For physiological analysis, means were compared by one-way analysis of variance using SPSS 17.0 software (SPSS, Chicago, IL, United States), and three biological replicates were used. For proteomics data, four biological replicates were analyzed by Student *t*-test. A *p*-value smaller than 0.05 was considered to be statistically significant.

## Results

### Changes of Morphology and RWC in Leaves Under Heat Stress

**FIGURE 1:**
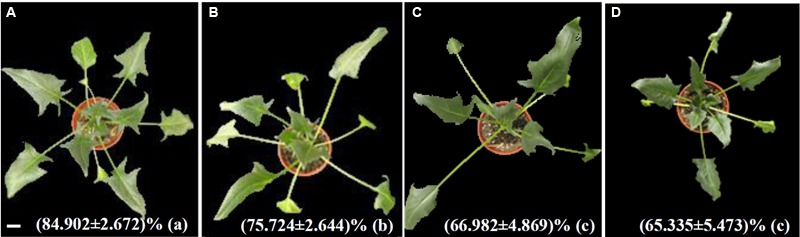
Morphological characteristics of spinach after 37°C treatment for 0 h **(A)**, 24 h **(B)**, 48 h **(C)**, and 72 h **(D)**. Relative water content was presented as means ± standard deviation at bottom of each morphological images. Lower case letters in the bracket indicate significant differences among different treatments (*p* < 0.05), Bar = 2.9 cm.

The morphology and RWC of spinach leaves were obviously affected by heat stress treatment. Heat stress-treated plants exhibited slight withering at 24 HHT compared to control plants (**Figures 1A,B**). The number of withered leaves increased, and the withering was more serious at 48 and 72 HHT (**Figures 1C,D**). The RWC decreased by 11, 21, and 23% after heat treatment for 24, 48, and 72 HHT, respectively (**Figure 1**). This implied that cellular osmotic potential changed along with the heat-reduced water potential caused by the decrease of RWC. Here, it should be noted that FW was used to normalize the contents of MDA, proline, soluble sugar, H_2_O_2_, AsA, and GSH in some previous studies, which might cause a bias given that the stress-caused decrease of RWC. Although we also used FW in this study, DW might be a better denominator to use in the future.

### Changes of Photosynthetic Characteristics in Response to Heat Stress

**FIGURE 2:**
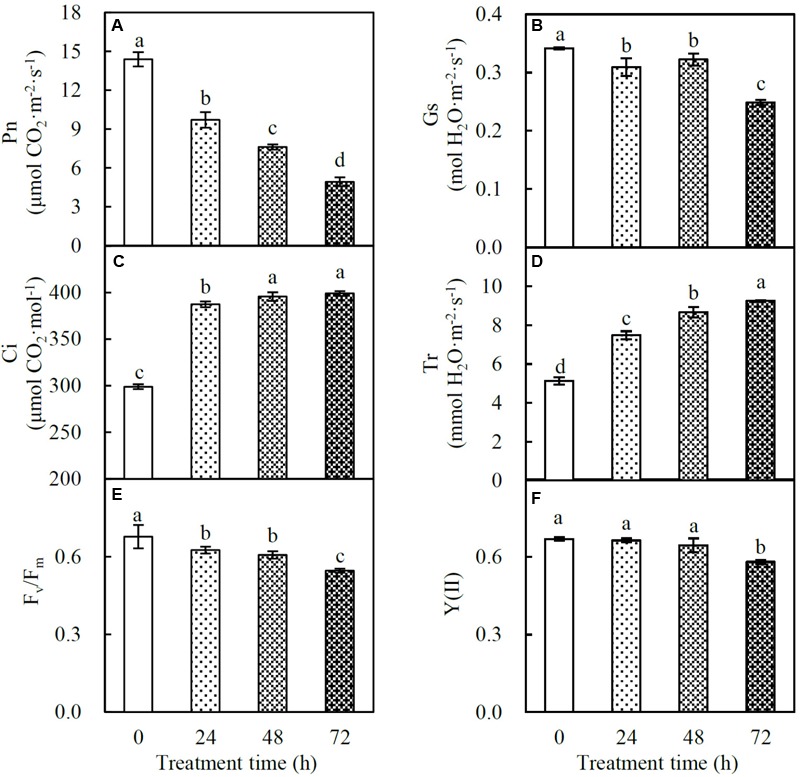
Photosynthetic characteristics in spinach under heat stress. **(A)** Net photosynthetic rate (Pn); **(B)** stomatal conductance (Gs); **(C)** intercellular CO_2_ concentration (Ci); **(D)** transpiration rate (Tr); **(E)** F_v_/F_m_; **(F)** Y(II). Lower case letters indicate significant differences among different treatments (*p* < 0.05).

Photosynthesis of spinach is very sensitive to heat stress. The Pn decreased gradually in the course of the heat treatment (**Figure 2A**). Gs also decreased in response to the heat stress. There was no significant difference between plants at 24 and 48 HHT, and a 27% decrease in Gs compared to control plants was observed at 72 HHT (**Figure 2B**). Besides these, Ci and Tr increased significantly under heat stress (**Figures 2C,D**). The parameters F_v_/F_m_ and Y(II) represent the maximal and effective quantum yield of PS II photochemistry, respectively. F_v_/F_m_ decreased gradually during the treatment (**Figure 2E**), while Y(II) was significantly reduced at 72 HHT (**Figure 2F**).

### Osmolyte Content and Cell Membrane Integrity

**FIGURE 3:**
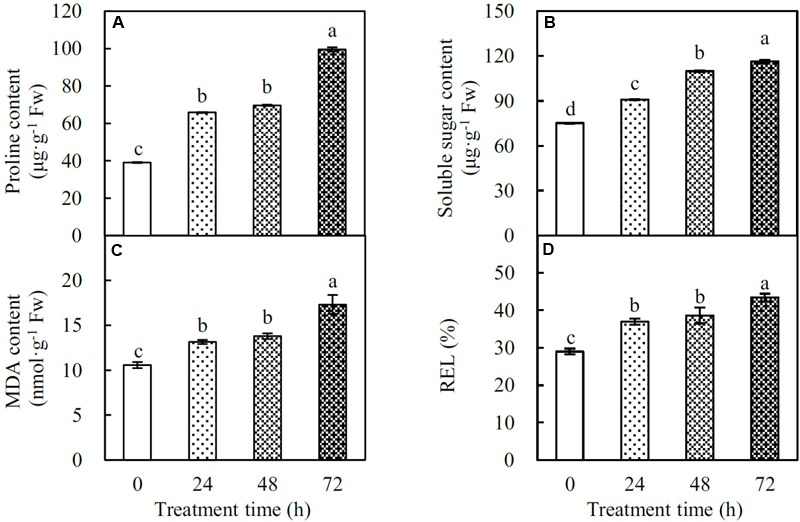
Osmolyte contents and cell membrane integrity indexes in spinach under heat stress. **(A)** Proline content; **(B)** soluble sugar content; **(C)** malondialdehyde (MDA) content; **(D)** relative electrolyte leakage (REL). Lower case letters indicate significant differences among different treatments (*p* < 0.05).

Proline and soluble sugars are key osmolytes facilitating plant stress adaptation. In spinach leaves, the proline content gradually increased by 1.7-, 1.8-, and 2.5-fold at 24, 48, and 72 HHT, respectively. Similarly, the soluble sugar content also increased by 1.2-, 1.5-, and 1.5-fold, respectively, under the above conditions (**Figures 3A,B**). In addition, MDA and REL were considered as two indexes of heat-induced cell membrane damage. Our data showed that MDA content significantly increased by 1.2-, 1.3-, and 1.6-fold, and REL increased by 1.3-, 1.3-, and 1.5-fold at 24, 48, and 72 HHT, respectively (**Figures 3C,D**), indicating the loss of membrane integrity in heat-stressed spinach leaves.

### Effects of Heat Stress on Antioxidant Enzyme Systems

**FIGURE 4:**
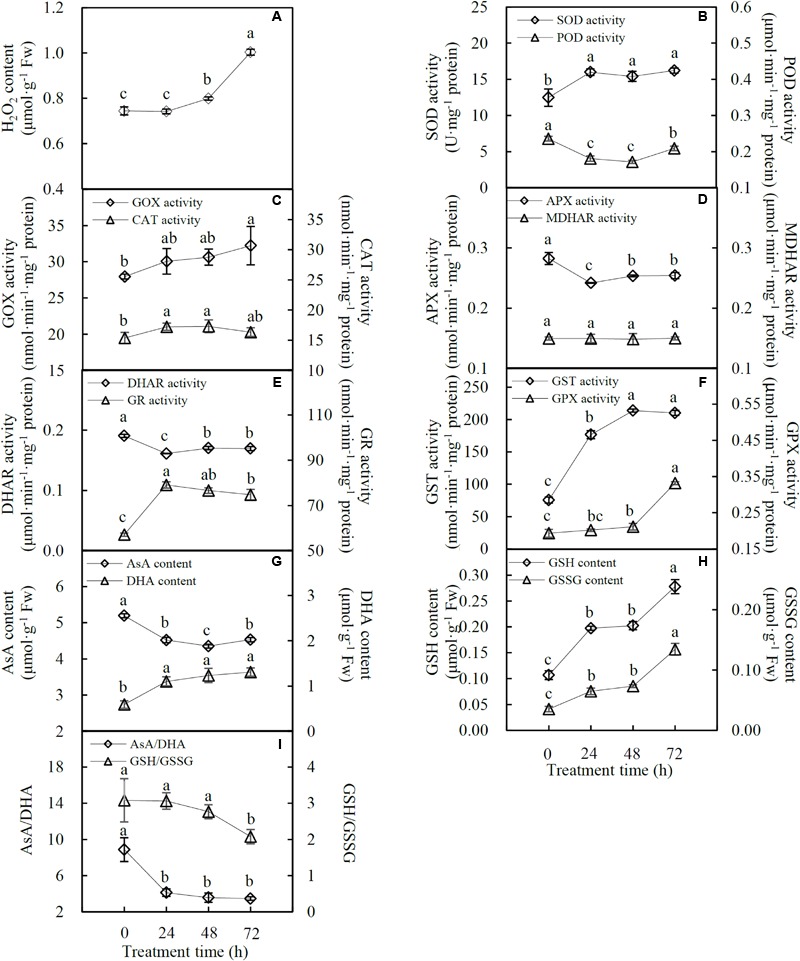
H_2_O_2_ content, antioxidant enzyme activities and antioxidant contents in spinach under heat stress. **(A)** H_2_O_2_ content; **(B)** activities of superoxide dismutase (SOD) and peroxidase (POD); **(C)** glycolate oxidase (GOX) and catalase (CAT) activities; **(D)** activities of ascorbate peroxidase (APX) and monodehydroascorbate reductase (MDHAR); **(E)** activities of dehydroascorbate reductase (DHAR) and glutathione reductase (GR); **(F)** activities of glutathione S-transferase (GST) and glutathione peroxidase (GPX); **(G)** contents of ascorbate (AsA) and dehydroascorbate (DHA); **(H)** contents of glutathione (GSH) and oxidized glutathione (GSSG); and **(I)** ratios of AsA/DHA and GSH/GSSG. Lower case letters indicate significant differences among different treatments (*p* < 0.05).

The H_2_O_2_ content was not affected at 24 HHT, while it significantly increased by 1.1- and 1.3-fold at 48 and 72 HHT, respectively (**Figure 4A**). The activities of 10 antioxidant enzymes were also affected by heat stress. SOD activity significantly increased by 1.3-, 1.2-, and 1.3-fold at 24, 48, and 72 HHT, respectively (**Figure 4B**). In contrast, POD activity decreased by 0.8-, 0.7-, and 0.9-fold along with the increase of heat treatment duration (**Figure 4B**). GOX activity showed a significant increase of 1.2-fold at 72 HHT, while CAT activity increased by 1.1-fold at both 24 and 48 HHT, and changed to the same level as the control sample at 72 HHT (**Figure 4C**). In the AsA-GSH cycle, APX activity decreased by 0.9-fold, and DHAR activity decreased by 0.8-, 0.9-, and 0.9-fold under three heat stress conditions, respectively. However, MDHAR activity was not altered, and GR activity significantly increased by 1.4-, 1.3-, and 1.3-fold at 24, 48, and 72 HHT, respectively (**Figures 4D,E**). In addition, GST activity was gradually enhanced by 2.3-, 2.8-, and 2.8-fold in the course of the heat stress treatment (**Figure 4F**). GPX activity was not affected at 24 HHT, and increased by 1.1- and 1.7-fold at 48 and 72 HHT, respectively (**Figure 4F**). Heat stress also led to the changes of antioxidant content, including AsA, DHA, GSH, and GSSG. AsA content decreased by 0.9-, 0.8-, and 0.9-fold, while DHA content increased by 1.9-, 2.1-, and 2.2-fold at 24, 48, and 72 HHT, respectively, leading to the significant decrease of AsA/GSH ratio (**Figures 4G,I**). In addition, the contents of GSH and GSSG showed a similar heat stress-increased trend (**Figure 4H**). GSH content increased by 1.9-, 1.9-, and 2.6-fold, and GSSG content increased by 1.8-, 2.1-, and 3.1-fold under the three heat stress conditions. However, the GSH/GSSG ratio did not change at 24 and 48 HHT, and decreased by 0.9-fold at 72 HHT (**Figure 4I**).

### Heat Stress-Responsive Proteome in Spinach Leaves

The HRP abundance pattern in spinach leaves was analyzed using iTRAQ-based quantitative proteomic approach. In total, 2,205 protein species in leaves were identified in at least three independent biological replicates, 911 of which were defined as HRPs using a threshold of significance of *p* < 0.05, and with a fold change >1.2 in protein abundance. Among them, 618 protein species increased and 281 decreased under at least one of the heat stress conditions, while the 12 remaining showed differently changed patterns in response to the heat stress treatments (Supplementary Figure S2).

**FIGURE 5:**
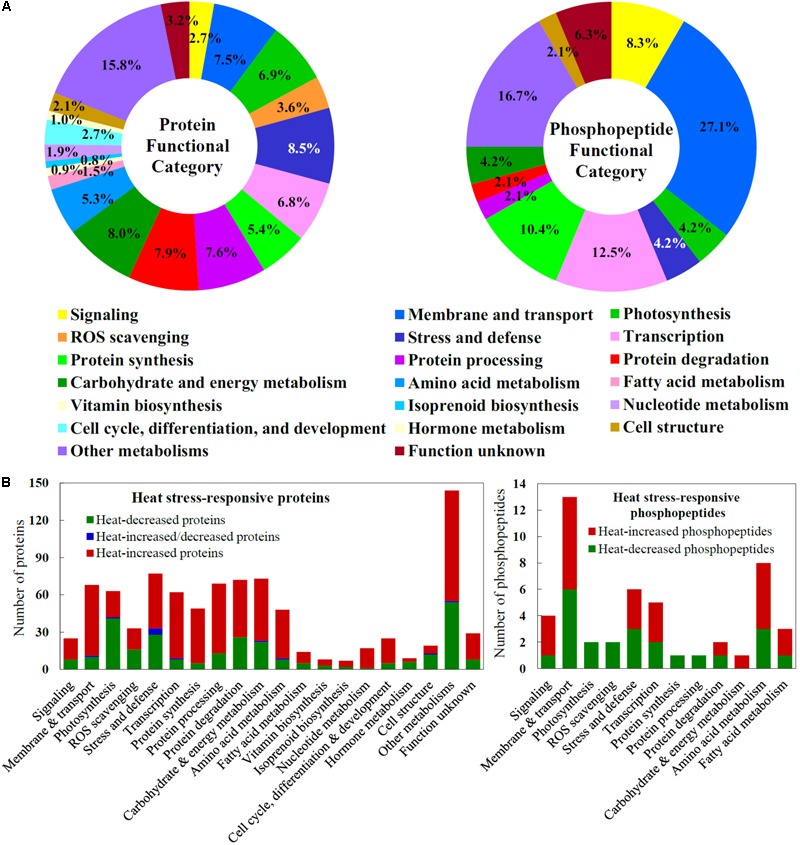
Functional categorization and abundance patterns of heat stress-responsive proteins and phosphopeptides in spinach leaves. **(A)** A total of 911 heat stress-responsive proteins were classified into 20 functional categories, and a total of 48 heat stress-responsive phosphopeptides were classified into 12 functional categories. The percentage of proteins in each functional category is shown in the pie. **(B)** Abundance patterns of heat stress-responsive proteins and phosphopeptides in each functional category.

The HRPs were classified into 20 functional categories (**Figure 5A** and Supplementary Table S1). The majority of the heat stress-increased proteins indicated that active signaling and metabolic networks have been initiated in spinach leaves to cope with the heat stress (**Figure 5B**). For example, 17 out of 25 (68%) HRPs involved in diverse signaling pathways were heat stress-increased, and they mainly functioned in lipid signaling, calcium signal transmitting, phosphorylation cascades, and 14-3-3-mediated signaling pathways. Importantly, 84% proteins in membrane and transport pathways were heat stress-increased, and they include proteins for endomembrane trafficking, Ran GTPase-mediated nuclear trafficking, as well as water, ion, and metabolite transporters. Also, 52% heat stress-increased proteins were involved in ROS scavenging pathway, e.g., SOD, CAT, GST, Prx, Grx, and Trx, and 57% stress and defense-related proteins (e.g., aldehyde dehydrogenase, aldo/keto reductase, lactoylglutathione lyase, late embryogenesis abundant protein, lectin, and jasmonate-induced protein) were heat stress-increased in leaves. Furthermore, the majority of HRPs in each group were heat stress-increased, including HRPs in transcription (85%), protein synthesis (90%), protein processing (81%), and protein degradation (64%). This suggested that gene expression and protein turnover were enhanced in response to heat stress. Also, heat stress-increased proteins took a major part in various metabolic pathways, including those of carbohydrate/energy (68%), amino acids (81%), fatty acids (64%), vitamins biosynthesis (63%), isoprenoids biosynthesis (71%), nucleotides (94%), as well as cell cycle, differentiation and development (80%). In contrast, most proteins involved in photosynthesis (65%), hormone metabolism (67%), and cell structure (63%) were heat stress-decreased, indicating these pathways were probably inhibited under heat stress (**Figure 5B** and Supplementary Table S1).

### Heat Stress-Responsive Phosphoproteome in Spinach Leaves

**FIGURE 6:**
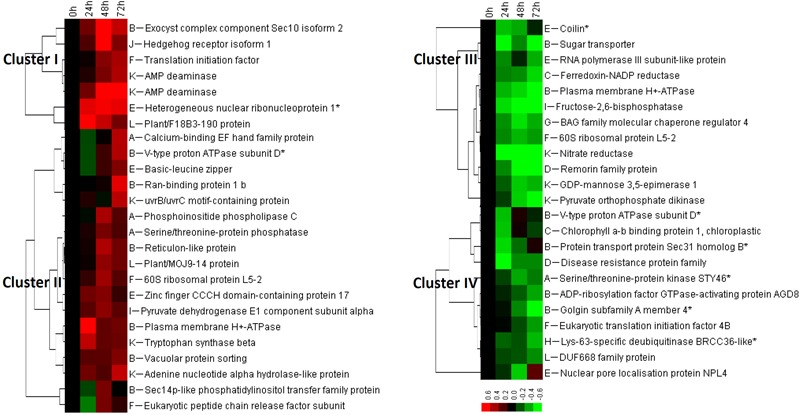
Cluster analysis of phosphorylation level change patterns of heat stress-responsive phosphopeptides in spinach leaves. The columns represent different treatment conditions including 37°C treatment for 0, 24, 48, and 72 h, and the rows represent individual phosphopeptides. The scale bar indicates log (base 2) transformed relative phosphorylation levels of phosphopeptides. Four main clusters are shown in the figure, and functional categories are indicated by upper case letters (A, signaling; B, membrane and transport; C, photosynthesis; D, stress and defense; E, transcription; F, protein synthesis; G, protein processing; H, protein degradation; I, carbohydrate and energy metabolism; J, cell cycle, differentiation, and development; K, other metabolisms; L, function unknown).

In spinach leaves, 111 phosphopeptides representing 103 phosphoproteins were identified using a TiO_2_ enrichment-based proteomics approach. Among them, 48 heat stress-responsive phosphopeptides representing 45 phosphoproteins (belonging to 12 functional categories) were detected with more than 1.2-fold changes (*p* < 0.05) of phosphorylation level, including 25 heat stress-increased and 23 heat stress-decreased phosphopeptides (**Figures 5A,B** and Supplementary Table S2). Hierarchical clustering analysis of these phosphopeptides generated four main clusters (**Figure 6**). The phosphorylation levels of seven phosphopeptides involved in six processes in Cluster I increased under two or three heat stress conditions. Cluster II included 18 phosphopeptides, whose phosphorylation levels increased under one heat stress condition. The corresponding proteins were mainly involved in signaling, membrane and transport, transcription, protein synthesis, and other metabolisms. Additionally, in Cluster III, 12 phosphopeptides showed heat stress-decreased phosphorylation levels under two or three heat stress conditions, and they were mainly responsible for membrane and transport, transcription, and other metabolisms. Furthermore, cluster IV included 11 phosphopeptides with heat stress-decreased phosphorylation levels under one of the three treatment conditions, and membrane and transport proteins were most abundant in this cluster (**Figure 6**).

### Three-Dimensional Structure Modeling of Heat Stress-Responsive Phosphoproteins

**FIGURE 7:**
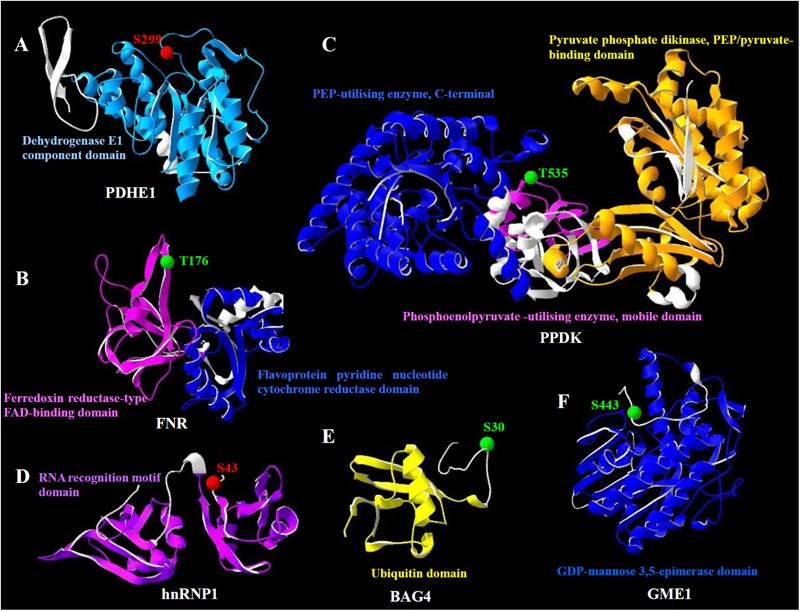
Homology models of the heat stress-responsive phosphoproteins from spinach leaves in three dimensions. **(A)** Pyruvate dehydrogenase E1 component subunit alpha (PDHE1); **(B)** ferredoxin-NADP reductase (FNR); **(C)** pyruvate orthophosphate dikinase (PPDK); **(D)** heterogeneous nuclear ribonucleoprotein 1 (hnRNP1); **(E)** BAG family molecular chaperone regulator 4 (BAG4); **(F)** GDP-mannose 3,5-epimerase 1 (GME1). The phosphorylation sites are shown with balls in red (increased phosphorylation level) and green (decreased phosphorylation level). Domains from the phosphoprotein and corresponding names are highlighted in the same color. Detailed information for these homology models can be found in Supplementary Table S3.

The molecular structure of heat stress-responsive phosphoproteins was predicted for better understanding the biochemical functions of the protein phosphorylation during the heat stress response. In total, 17 statistically acceptable homology models were built through the SWISS-MODEL. Among them, the phosphorylation sites of six phosphoproteins were located within the three-dimensional structure models (**Figure 7**), while models for the rest 11 phosphoproteins did not contain the corresponding phosphorylation sites. The phosphorylation sites and numbers of helices and beta sheets were presented in the three-dimensional models (**Figure 7** and Supplementary Table S3).

The heat stress-increased phosphorylation site of pyruvate dehydrogenase E1 component subunit alpha occurred on Ser299, which was located in the dehydrogenase E1 component domain (**Figure 7A**). However, the phosphorylation levels of ferredoxin-NADP reductase and pyruvate orthophosphate dikinase decreased under heat stress. Their phosphorylation sites, Thr176 and Thr535, were located in the ferredoxin reductase-type FAD-binding domain and phosphoenolpyruvate-utilizing enzyme mobile domain, respectively (**Figures 7B,C**). Also, the phosphorylation level of heterogeneous nuclear ribonucleoprotein 1 was heat stress-increased. However, the phosphorylation site Ser43 was not located in its functional domain (**Figure 7D**). Similarly, the phosphorylation sites of BAG family molecular chaperone regulator 4 and GDP-mannose 3,5-epimerase 1, Ser30 and Ser443 were heat stress-decreased and not localized in their functional domains (**Figures 7E,F**).

## Discussion

### Diverse Calcium-Mediated Signaling Pathways Are Induced in the Heat-Tolerant Spinach

**FIGURE 8:**
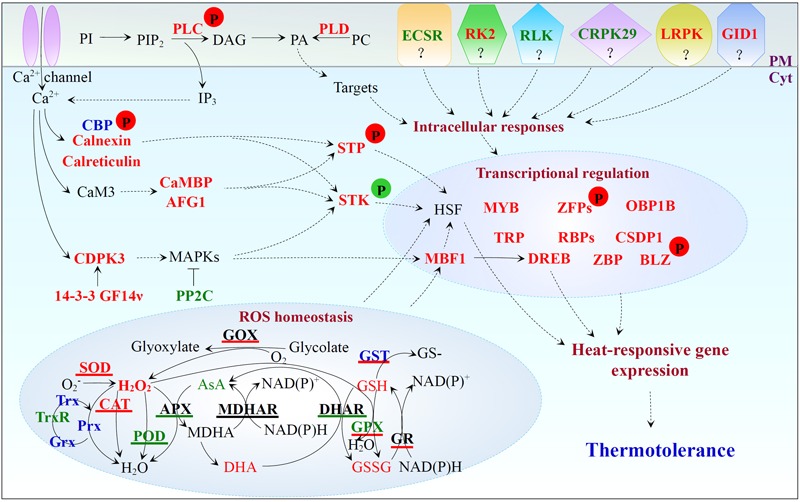
Heat stress-responsive signal transduction, ROS homeostasis, and transcriptional regulation in spinach. Protein names in red and green represent increased and decreased protein abundances under heat stress, respectively. Protein names in blue indicate that the abundance change patterns of multiple protein species in each protein family were varied. Antioxidant enzymes with red, green, and black underline indicate the activities are induced, reduced, and stable under heat stress, respectively. Substrate names with red and green indicate the heat-increased and heat-decreased contents, respectively. Arrows with solid and dashed lines represent direct stimulation/single-step reaction and indirect stimulation/multi-step reaction, respectively. The “T” shape line represents inhibition. A “P” in red and green circles indicates increased and decreased phosphorylation levels of the corresponding proteins, respectively. 14-3-3 GF14ν, 14-3-3-like protein GF14ν; AFG1, AFG1-like ATPase; APX, ascorbate peroxidase; AsA, ascorbate; BLZ, basic-leucine zipper; CaM, calmodulin; CaMBP, calmodulin-binding protein; CAT, catalase; CBP, calcium-binding EF hand family protein; CDPK, calcium-dependent protein kinase; CRPK29, cysteine-rich receptor-like protein kinase 29; CSDP1, cold shock domain protein 1; Cyt, cytosol; DAG, diacylglycerol; DHA, dehydroascorbate; DHAR, dehydroascorbate reductase; DREB, dehydration-responsive element-binding; ECSR, extracellular calcium sensing receptor; GID1, gibberellin receptor GID1; GOX, glycolate oxidase; GPX, glutathione peroxidase; GR, glutathione reductase; Grx, glutaredoxin; GSH, glutathione; GSSG, oxidized glutathione; GST, glutathione S-transferase; HSF, heat shock transcription factor; IP3, D-*myo*-inositol 1,4,5-tris-phosphate; LRPK, leucine-rich repeat receptor-like protein kinase; MAPK, mitogen-activated protein kinase; MBF, multiprotein bridging factor; MDHAR, monodehydroascorbate reductase; OBP1B, oligouridylate-binding protein 1B; PA, phosphatidic acid; PC, phosphatidylcholine; PI, phosphatidylinositol; PIP2, phosphatidylinositol-4,5-bisphosphate; PLC, phospholipase C; PLD, phospholipase D; PM, plasma membrane; POD, peroxidase; Prx, peroxiredoxin; RBP, RNA-binding protein; RK2, receptor kinase 2; RLK, receptor-like kinase; ROS, reactive oxygen species; SOD, superoxide dismutase; STK, serine/threonine-protein kinase; STP, serine/threonine-protein phosphorylase; TRP, tetratricopeptide repeat-like superfamily protein; Trx, thioredoxin; TrxR, thioredoxin reductase; ZBP, zinc-binding protein; ZFP, zinc finger family protein.

High temperature increases plasma membrane (PM) fluidity, leading to a transient calcium influx through calcium channels on PM (Mittler et al., 2012). High temperature also induces lipid signaling pathway to trigger the release of calcium from internal stores. In heat-stressed spinach, phospholipase D (PLD) and phospholipase C (PLC)-like phosphodiesterase, as well as the phosphorylation level of PLC increased (**Figure 8** and Supplementary Table S1). Consistently, heat stress-induced phosphorylation of PLC was reported in maize leaves (Hu et al., 2015). Heat stress-increased PLD and PLC facilitated the accumulation of lipid signaling molecules phosphatidic acid and D-*myo*-inositol 1,4,5-tris-phosphate to trigger the release of calcium from internal stores, which may activate diverse calcium-dependent signaling pathways (Mishkind et al., 2009; Zheng et al., 2012). We found calcium-dependent protein kinase 3 (CDPK3), multiprotein bridging factor 1 (MBF1), dehydration-responsive element-binding (DREB) transcription factor, and 14-3-3 GF14ν were all heat stress-increased, except for protein phosphatase 2C (PP2C) (**Figure 8** and Supplementary Table S1). Calcium may directly activate CDPKs and then induce multiple mitogen-activated protein kinases (MAPKs) (Sangwan et al., 2002), leading to the activation of downstream transcription factors (i.e., MBF1, DREB, and heat shock transcription factors (HSFs)) for regulation of the heat stress-responsive genes (Suzuki et al., 2011). Previous studies have reported that *MBF1c* expression was up-regulated in heat-stressed grape leaves (Jiang et al., 2017), and PP2C negatively regulated the MAPK pathway (Rodriguez, 1998). Thus, the heat-decreased PP2C would facilitate the activation of calcium-mediated MAPK cascades. In addition, 14-3-3 proteins function as major regulators of a wide range of proteins involved in signaling, transcription activation, primary metabolism, and stress defense (Roberts et al., 2002). For example, it has been reported that the kinetics of cold-induced accumulation of two 14-3-3 genes (i.e., *RCI1/RCI1A* and *RCI2/RCI1B*) was correlated with the increase of plant freezing tolerance (Jarillo et al., 1994; Abarca et al., 1999). Overexpression of a 14-3-3 encoding gene *GF14λ* confers plant tolerance to drought stress (Yan et al., 2004). How the three heat stress-induced 14-3-3-like proteins activate CDPK and/or MAPK signaling pathways in response to heat stress deserves further investigation.

In spinach leaves, the heat stress-altered abundances and heat-induced phosphorylation of CBPs implied that the increased calcium enhanced the activities of CBPs (**Figure 8** and Supplementary Table S1). CBPs together with heat-increased calnexin and calreticulin may promote the intracellular calcium homeostasis and signaling, as well as protein folding in spinach leaves (**Figure 8** and Supplementary Table S1; Jia et al., 2008; Sarwat and Naqvi, 2013; Garg et al., 2015). In addition, the heat stress-induced calcium would bind to the calmodulin 3 (CaM3) (Zhang et al., 2009) to activate calcium/CaM-binding protein kinases or phosphorylases to modulate HSFs activity (Reddy et al., 2011). In this study, two heat stress-increased CaM target proteins (CaM-binding protein and AFG1-like ATPase) implied their possible involvement in calcium/CaM signaling events. It was reported that the phosphorylation level of serine/threonine-protein kinase wnk4-like was increased in maize leaves under heat treatment (Hu et al., 2015). In this study, heat stress induced the abundance of a serine/threonine-protein kinase STY46 in spinach leaves, but decreased its phosphorylation level (**Figure 8** and Supplementary Table S1). Such a coordination of protein level and post-translational modification level is interesting. We also found heat stress decreased the abundances of extracellular calcium sensing receptor, receptor-like kinase, and cysteine-rich receptor-like protein kinase 29, but increased the abundances of receptor kinase 2, leucine-rich repeat receptor-like protein kinase, and gibberellin receptor GID1 in spinach leaves (**Figure 8** and Supplementary Table S1). The biological implications of these changes are not known.

### Active Endomembrane Trafficking and Cross-Membrane Transport Are Crucial for Spinach Heat Responses

**FIGURE 9:**
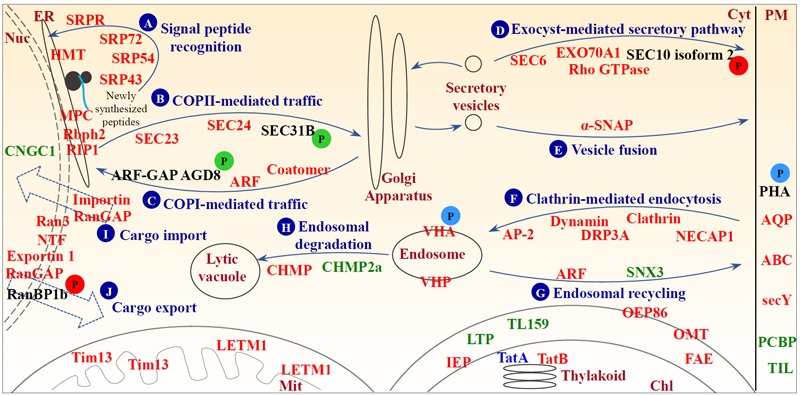
Schematic representation of heat stress-responsive proteins involved in membrane and transport. **(A)** Signal peptide recognition; **(B)** COPII-mediated traffic; **(C)** COPI-mediated traffic; **(D)** exocyst-mediated secretory pathway; **(E)** vesicle fusion; **(F)** clathrin-mediated endocytosis; **(G)** endosomal recycling; **(H)** endosomal degradation; **(I)** cargo import; and **(J)** cargo export. Protein names in red and green represent increased and decreased protein abundances under heat stress, respectively. Protein names in blue indicate differential abundance change patterns of different protein species in the same protein family. A “P” in red and green circles indicates increased and decreased phosphorylation levels of the corresponding proteins, respectively. A “P” in blue circles indicates differential phosphorylation level changes of different phosphopeptides belonging to the corresponding proteins. ABC, ATP-binding cassette transporter; ANX, annexin; AP-2, adaptor protein complex AP-2; AQP, aquaporin; ARF, ADP-ribosylation factor; ARF-GAP, ADP-ribosylation factor GTPase-activating protein; Chl, chloroplast; CHMP, charged multivesicular body protein; CNGC1, potassium/sodium hyperpolarization-activated cyclic nucleotide-gated channel 1; Cyt, cytosol; DRP, dynamin-related protein; ER, endoplasmic reticulum; FAE, fatty acid export 3, chloroplastic; GAP, GTPase-activating protein; GEF, guanine exchange factor; HMT, heavy metal transport/detoxification superfamily protein; IEP, non-green plastid inner envelope membrane protein; LETM1, LETM1 and EF-hand domain-containing protein 1; LTP, lipid transfer protein; Mit, mitochondrion; MPC, endoplasmic reticulum membrane protein complex subunit-like protein; NECAP, adaptin ear-binding coat-associated protein; NTF, nuclear transport factor; Nuc, nucleus; OEP86, chloroplast outer envelope protein 86; OMP, mitochondrial outer membrane protein porin-like; OMT, 2-oxoglutarate/malate translocator; PCBP, plasma-membrane associated cation-binding protein 1; PHA, plasma membrane H^+^-ATPase; PHB, prohibitin; PM, plasma membrane; RanBP, Ran-binding protein; RanGAP, Ran GTPase activating protein; Rbph2, ribophorin 2 family protein; RIP1, reticulon-4-interacting protein 1; SAR1, secretion-associated and ras-superfamily-related 1; secY, protein translocase secY subunit; SNX, sorting nexin; SRP, signal recognition particle protein; SRPR, signal recognition particle protein receptor; TatA, sec-independent protein translocase protein TATA; TatB, sec-independent protein translocase protein TATB; TIL, temperature-induced lipocalin; Tim13, mitochondrial import inner membrane translocase subunit Tim13; TL159, translocase of chloroplast 159; VHA, vacuolar H^+^-ATPase; VHP, vacuolar H^+^-pyrophosphatase; α-SNAP, alpha-soluble NSF attachment protein.

In this study, 22 out of 24 membrane trafficking-related proteins were heat-increased, which implied that an active endomembrane trafficking was critical for spinach heat responses (Singh and Jürgens, 2017; Supplementary Table S1). Three heat stress-increased signal recognition particle proteins and a membrane-associated signal recognition particle receptor would facilitate recognizing the signal peptides of newly synthesized proteins for endoplasmic reticulum (ER) targeting under heat stress (**Figure 9A** and Supplementary Table S1; Jürgens, 2004). Additionally, the increases of five HRPs indicate both COPII vesicle-mediated ER-Golgi anterograde transport and COPI vesicle-mediated Golgi-ER retrograde traffic were activated in spinach leaves (**Figures 9B,C** and Supplementary Table S1). Transport vesicles are formed on donor membranes by the coordinated action of small GTPases of ADP-ribosylation factor (ARF)/secretion-associated and ras-superfamily-related 1 (SAR1) family protein, their guanine-nucleotide exchange factors, GTPase-activating proteins (GAPs) and coat proteins. During anterograde transport, SEC23 with SAR1-specific GTPase-activating activity and SEC24 responsible for cargo selection were heat stress-increased. However, the phosphorylation level of SEC31, which functions as an outer coat protein to augment GAP activity on SAR1 GTPase, decreased under the heat stress (**Figure 9B** and Supplementary Table S1; Jürgens, 2004). In retrograde traffic, the abundances of ARF GTPase and two coatomer subunits increased, and the phosphorylation level of ARF-GAP AGD8 decreased (**Figure 9C** and Supplementary Table S1). This implies that the activity of ARF GTPase might be enhanced to activate vesicle trafficking between Golgi and ER, because ARF-GAP ASAP1 can be directly phosphorylated by proline-rich tyrosine kinase 2, resulting in a reduction of ARF GTPase activity (Kruljac-Letunic et al., 2003).

Since the abundance of exocyst complex components (i.e., SEC6 and EXO70 family protein A1) and Rho GTPase, and the phosphorylation level of SEC10 isoform 2 were heat stress-increased (**Figure 9D** and Supplementary Table S1), exocyst-mediated secretory pathways may be enhanced. It has been reported that the phosphorylation of SEC5 causes dissociation of exocyst from the small GTPase, resulting in the release of the vesicle from the exocyst for fusing with the target membrane (Chen X. W. et al., 2011). Since the exocyst complex mediates the tethering between secretory vesicles and the target membrane, and is regulated by Rab and Rho GTPases, our findings indicated that the function of exocyst complex would be enhanced and thereby facilitate the regulation of cell polarity and morphogenesis (Žárský et al., 2013). In addition, heat stress-increased α-soluble *N*-ethylmaleimide-sensitive factor association protein implies the acceleration of protein transport and exchange via vesicle fusion in the heat-stressed leaves (**Figure 9E** and Supplementary Table S1).

Several heat stress-increased proteins are involved in clathrin coat assembly during endocytosis (i.e., clathrin heavy chain, adaptor protein complex AP-2, and adaptin ear-binding coat-associated protein 1) and membrane scission of nascent endocytic vesicles (i.e., dynamin and dynamin-related protein 3A) (**Figure 9F** and Supplementary Table S1; Ritter et al., 2003; Paez Valencia et al., 2016). These results indicate clathrin-mediated endocytosis were heat-increased, and thereby regulated both abundances and distributions of important molecules at the cell surface (e.g., signaling receptors and transporters) under heat stress. On the other hand, the process of recycling of transmembrane receptors and other integral proteins from endosomes back to the PM mainly mediated by small GTPases and the retromer complex was also altered in spinach leaves under the heat stress (**Figure 9G** and Supplementary Table S1). In addition, proteins targeted for degradation through ubiquitination need to be sorted into endosomal vesicles by the endosomal sorting complex required for transport (ESCRT) machinery for the degradation in the lytic vacuole (Paez Valencia et al., 2016). Here two core subunits of ESCRT-III were affected by heat stress (**Figure 9H** and Supplementary Table S1), indicating endosomal-mediated protein degradation was regulated to some extent by the heat stress. Moreover, the heat stress may have induced nucleo-cytoplasmic trafficking through Ran GTPase-dependent pathways in spinach leaves, as seven proteins involved in Ran-mediated nuclear trafficking, including GTP-binding nuclear protein Ran 3, RanGAP, nuclear transport factor, exportin 1, and three importins, as well as phosphorylation level of Ran-binding protein 1b (RanBP1b) were heat stress-increased (**Figures 9I,J** and Supplementary Table S1; Quimby and Dasso, 2003; Zang et al., 2010). Among them, members of Ran GTPases, such as Ran 1A, Ran 1B, and Ran 2, have been proved to be heat-increased in heat-tolerant *A. scabra* and heat-sensitive *A. stolonifera* (Sangwan et al., 2002; Xu and Huang, 2008), as well as wheat grain (Majoul et al., 2004). In rice, OsRAN2 is known to regulate cold tolerance by maintaining cell division through promoting the normal export of intranuclear tubulin at the end of mitosis (Chen N. et al., 2011). The heat-responsive functions and mechanisms of Ran 3 and other aforementioned proteins in the nucleo-cytoplasmic trafficking have not been reported.

Several transporters responsible for water, ion, and metabolite transport were heat stress-increased, such as aquaporins, vacuolar H^+^-ATPase, H^+^-pyrophosphatase, and ATP-binding cassette (ABC) transporters (Supplementary Table S1). Aquaporins are known to be associated with heat stress (Christou et al., 2014; Obaid et al., 2016). The vacuolar H^+^-ATPase and H^+^-pyrophosphatase are required for membrane potential, pH homeostasis, and secondary solute transport during adaptation to a variety of abiotic stresses (Gaxiola et al., 2007). Heat stress induced *vha-A, vha-C*, and *vha-c*, but reduced other *vha* transcripts including *vha-a* and *vha-H* in *Mesembryanthemum crystallinum* leaves (Kluge et al., 2003), and overexpression of vacuolar H^+^-pyrophosphatase confers tolerance to heat stress (Dong et al., 2011; Yoon et al., 2013). In addition, ABC transporters shuttle diverse substrates (e.g., lipids, phytohormones, carboxylates, heavy metals, chlorophyll catabolites, and xenobiotic conjugates) across biological membranes, and they play important roles in cellular detoxification, growth and development, and pathogen defense (Kretzschmar et al., 2011; Jiang et al., 2017).

### Photosynthesis Is Inhibited Under Prolonged Heat Stress

**FIGURE 10:**
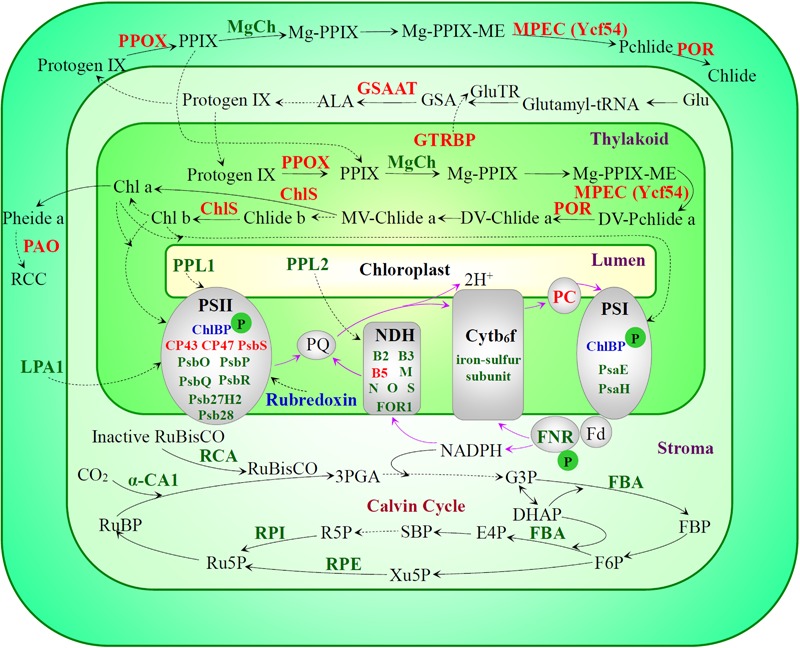
Schematic representation of heat stress-responsive photosynthesis in spinach leaves. Please refer to **Figure 8** legend for color scheme of the protein names, and solid and dashed lines. Arrows with purple lines represent the photosynthetic electron transfer direction. A “P” in green circles indicates decreased phosphorylation levels of the corresponding proteins. 3PGA, 3-phosphoglycerate; ALA, 5-aminolevulinic acid; Chl, chlorophyll; ChlBP, chlorophyll a/b binding protein; Chlide, chlorophyllide; ChlS, chlorophyll synthase; Cytb_6_f, cytochrome b_6_f complex; DHAP, dihydroxyacetone phosphate; DV-Chlide, divinyl chlorophyllide; DV-Pchlide, divinyl protochlorophyllide; E4P, erythrose-4-phosphate; F6P, fructose 6-phosphate; FBA, Fructose-bisphosphate aldolase; FBP, fructose-1,6-bisphosphate; Fd, ferredoxin; FNR, ferredoxin-NADP reductase; G3P, glyceraldehyde 3-phosphate; Glu, glutamate; GluTR, glutamyl-tRNA reductase; GSA, glutamate 1-semialdehyde; GSAAT, glutamate 1-semialdehyde aminotransferase; GTRBP, glutamyl-tRNA reductase binding protein; LPA, protein low PSII accumulation; MgCh, magnesium chelatase; Mg-PPIX, magnesium protoporphyrin IX; Mg-PPIX-ME, magnesium-protoporphyrin IX monomethyl ester; MPEC, magnesium-protoporphyrin IX monomethyl ester cyclase; MV-Chlide, monovinyl chlorophyllide; NDH, NAD(P)H dehydrogenase; PAO, pheophorbide a oxygenase; PC, plastocyanin; Pchlide, protochlorophyllide; Pheide, pheophorbide; POR, NADPH:protochlorophyllide oxidoreductase; PPIX, protoporphyrin IX; PPL, PsbP-like protein; PPOX, protoporphyrinogen oxidase; PQ, plastoquinone; Proto IX, protoporphyrin IX; Protogen IX, protoporphyrinogen IX; PS, photosystem; R5P, ribose-5-phosphate; RCA, ribulose bisphosphate carboxylase/oxygenase activase; RCC, red chlorophyll catabolite; RPE, ribulose-phosphate 3-epimerase; RPI, ribose-5-phosphate isomerase; Ru5P, ribulose-5-phosphate; RuBisCO, ribulose bisphosphate carboxylase/oxygenase; RuBP, ribulose-1,5-bisphosphate; SBP, sedoheptulose-1,7-bisphosphate; Xu5P, ketose xylulose-5-phosphate; α-CA, α-carbonic anhydrase.

In this study, photosynthetic parameters [i.e., Pn, Gs, F_v_/F_m_, and Y(II)] of spinach were obviously reduced with the increasing of heat treatment duration (**Figure 2**), indicating the photosynthetic apparatus was sensitive and impaired. The reduction of Gs may be attributed to low leaf water content caused by high Tr (**Figures 1, 2**; Mathur et al., 2014). The increased Tr is expected to protect leaves from heat stress by lowering leaf temperature, as one of the heat-adaptive strategies in spinach (Mathur et al., 2014). Additionally, it has been reported that Gs and Pn were inhibited in many heat-stressed plant species due to decreases of RuBisCO activity (Crafts-Brandner and Salvucci, 2002; Morales et al., 2003; Wahid et al., 2007; Mathur et al., 2014). In addition, it was found that the dark respiration rate increased in response to high temperature (Salvucci and Crafts-Brandner, 2004a), however, whether it was related with the heat-increased Ci needs further studies. Consistent with our findings (**Figure 10** and Supplementary Table S1), a number of transcriptomic and proteomic studies have revealed the heat stress-decreased photosynthesis in leaves of various plants (Wang X. et al., 2017), such as grape (Jiang et al., 2017), wheat (Lu et al., 2017), and soybean (Das et al., 2016). Among the 63 HRPs involved in photosynthesis, 14, 30, and 34 proteins showed decreased levels at 24, 48, and 72 HHT, respectively, indicating that the photosynthetic apparatus was affected severely with the prolonged heat-stress (Supplementary Table S1). The decreases of oxygen evolving complex (OEC) subunits (PsbO, PsbP, and PsbQ) indicated that PSII OEC might be dissociated under the heat stress (Wahid et al., 2007), similar to what took place in rice (Han et al., 2009), *C*. *spinarum* (Zhang et al., 2010), heat-tolerant *A*. *scabra*, and heat-sensitive *A. stolonifera* (Xu and Huang, 2010) under long-term (48 h, 120 h and 10 days) heat stress. Besides these, several other PSII proteins were heat-decreased at 48 and 72 HHT, such as PsbR involved in OEC assembly, Psb27-H2 responsible for C-terminal processing of D1 protein, Psb28 participating in CP47 assembly, PsbP-like protein 1 involved in PSII repair, PsbP-like protein 2 required for NAD(P)H dehydrogenase complex accumulation, as well as low PSII accumulation 1 and rubredoxin involved in PSII assembly and/or stability. In addition, PsaE and seven members of chloroplast NAD(P)H dehydrogenase decreased at 48 and/or 72 HHT (**Figure 10** and Supplementary Table S1). Similarly, 10-day-heat treatment resulted in the decrease of PSI reaction center subunit E in leaves of *A. scabra* and *A. stolonifera* (Xu and Huang, 2010). Moreover, cytochrome b_6_f complex and ferredoxin-NADP reductase involved in linear electron transfer also decreased in spinach under heat stress (**Figure 10** and Supplementary Table S1), as reported in wild rice (Scafaro et al., 2010) and soybean (Ahsan et al., 2010). All these results suggest that PSII, PSI, and electron transport in spinach were inhibited by prolonged heat stress, although it has been noted that moderately high temperatures could stimulate PSI activity and increase cyclic electron flow around PSI (Mathur et al., 2014).

The heat stress also decreased abundances of Calvin cycle enzymes, e.g., α-carbonic anhydrase 1 and RCA (**Figure 10** and Supplementary Table S1). Carbonic anhydrase was also found to be decreased by heat stress in grape (Liu G.T. et al., 2014) and soybean (Ahsan et al., 2010) at 6 and 12 HHT. RCA in *C*. *spinarum* increased at 48 HHT, but decreased after prolonged heat treatment (120 HHT) (Zhang et al., 2010). It has been proposed that inability of RCA to maintain RuBisCO in an active form attributes to the inhibition of net photosynthesis by moderate heat stress (Salvucci and Crafts-Brandner, 2004b). Therefore, the decreased RCA might be a limiting factor for photosynthesis in spinach under heat stress. In contrast to a previous report of heat stress decreased chlorophyll biosynthesis (Ashraf and Harris, 2013), our proteomic results showed that 9 out of 12 chlorophyll biosynthesis-related proteins were increased in the spinach leaves at 48 and 72 HHT (**Figure 10** and Supplementary Table S1), indicating chlorophyll biosynthesis might be enhanced to cope with decreased efficiency of electron transport and Calvin cycle activities under prolonged heat stress.

### Diverse ROS Scavenging Pathways are Employed for Heat Stress Responses

In spinach leaves, heat stress led to the accumulation of H_2_O_2_ (**Figure 4A**), which causes the autocatalytic peroxidation of membrane lipids thus leading to the impairment of membrane semi-permeability indicated by the increased MDA content and REL, respectively (**Figures 3C,D**). In order to protect cells from oxidative damage caused by the heat stress, spinach has developed both enzymatic and non-enzymatic detoxification systems to counteract ROS toxicity.

Diverse antioxidant enzyme pathways mediated by SOD, GOX, CAT, GPX, GST, GR, peroxiredoxin (Prx), glutaredoxin (Grx), and thioredoxin (Trx) were activated for ROS removal under heat stress. SOD acts as the first line of defense by catalyzing the dismutation of O_2_^-^ to O_2_ and H_2_O_2_ (Mittler et al., 2004). Heat stress significantly enhanced the SOD activity (**Figure 4B**), which is in agreement with the heat stress-increased SOD activity in leaves of maize (Zhao F. et al., 2016), poplar (Li et al., 2014), and wheat (Sairam et al., 2000; Kumar et al., 2012). Our proteomic results revealed that the abundances of SODs localized in chloroplast, mitochondrion, and cytoplasm of spinach leaves were also heat stress-increased (**Figure 8** and Supplementary Table S1). In addition, the activities of GOX and CAT were also significantly increased to facilitate the conversion of glycolate into glyoxylate and H_2_O_2_ (Dellero et al., 2016), as well as to remove of H_2_O_2_ generated in peroxisomes (Mittler et al., 2004). Also, the heat stress-increased activity and abundance of GST imply enhanced detoxification of electrophiles by GSH conjugation (Strange et al., 2001). Interestingly, the activity of GPX was also heat stress-increased, although the abundance of GPX decreased at 48 and 72 HHT (**Figure 8** and Supplementary Table S1). This can be explained by the independence of the normalized GPX activity on certain members of the GPX family (Bela et al., 2015). The heat stress-increased GPX was involved in reducing lipid hydroperoxides and H_2_O_2_ by using GSH as a direct electron donor (Noctor et al., 2002). Meanwhile, heat stress also increased GR activity to maintain the reduced pool of GSH in spinach (Gill et al., 2013). Additionally, the heat stress-increased Prx was also detected in spinach at 48 and/or 72 HHT (**Figure 8** and Supplementary Table S1) for improving the detoxification of H_2_O_2_ and other peroxides. For regenerating the active forms of Prxs and other target proteins in thiol/disulfide redox regulatory network (Bhatt and Tripathi, 2011), the abundance of one Grx and three Trxs were significantly increased at 48 and/or 72 HHT (**Figure 8** and Supplementary Table S1). Most of these heat stress-increased pathways have also been reported in other plant species. For example, the activities of SOD, CAT, and GR increased in leaves of maize (Zhao F. et al., 2016), wheat (Kumar et al., 2012), and poplar (Li et al., 2014) in response to heat stress. The heat stress-increased protein abundances of SOD, CAT, GST, Prx, and Trx were found in leaves of rice (Lee et al., 2007; Han et al., 2009), wheat (Majoul et al., 2003; Yang et al., 2011; Wang et al., 2015), soybean (Ahsan et al., 2010; Wang et al., 2012), *P*. *oleracea* (Yang et al., 2012), *A*. *scabra, A. stolonifera* (Xu and Huang, 2008), and poplar (Li et al., 2014). Moreover, genes encoding SOD, CAT, GST, GPX, Trx, and Grx were up-regulated by heat stress in leaves of spinach (Yan et al., 2016), carnation (Wan et al., 2015), *S*. *japonica* (Liu F. et al., 2014), and perennial ryegrass (Wang K. et al., 2017). All these results indicate that these pathways are conserved and essential in many plants. Although APX and DHAR pathways also increased in the plant species mentioned above, they were inhibited in spinach leaves under heat stress (**Figures 4D,E**). The POD pathway was inhibited in spinach leaves as evidenced by the heat stress-decreased POD activity (**Figure 4B**) and abundances of all the isoenzymes of POD (**Figure 8** and Supplementary Table S1). This result was consistent with previous findings in rice (Lee et al., 2007; Han et al., 2009), soybean (Ahsan et al., 2010), *A. scabra, A. stolonifera* (Xu and Huang, 2008), spinach (Yan et al., 2016), and perennial ryegrass (Wang K. et al., 2017).

Non-enzymatic antioxidants (i.e., GSH, proline, and soluble sugars) can be employed to scavenge free-radical in heat-stressed spinach (Wahid et al., 2007). Heat-induced GSH content can improve stress tolerance by maintaining the reduced state of diverse substances (e.g., proteins, α-tocopherol, and zeaxanthin) and serving as a substrate for GST and GPX (Cheng et al., 2015). Also, accumulation of certain compatible osmolytes (e.g., proline and soluble sugars) was also an effective strategy for dealing with oxidative stress induced by heat stress (Wahid et al., 2007). The heat stress-induced proline in tomato (Rivero et al., 2004), tobacco (Cvikrová et al., 2012), and poplar (Li et al., 2014), as well as heat stress-accumulated soluble sugars in sugarcane, have been shown to be important for heat stress adaptation (Wahid and Close, 2007). In spinach, the accumulation of proline and soluble sugars, as well as the increase of delta 1-pyrroline-5-carboxylate synthetase for proline biosynthesis (**Figure 8** and Supplementary Table S1) can help buffer cellular redox change and stabilize subcellular structures under heat stress (Hayat et al., 2012).

### Transcriptional Regulation and Protein Processing are Heat Stress-Induced

It is well-known that a number of transcription factors (e.g., HSF, DREB, MBF, and bZIP28) are heat-inducible (Wahid et al., 2007; Hua, 2009). In this study, we found that 53 of 62 heat stress-responsive transcription-related proteins were increased in spinach (Supplementary Table S1). They were in charge of chromosome/DNA modulation and repairing, transcriptional regulation, as well as RNA transport, processing, splicing, and cleavage. Clearly, transcriptional regulation was enhanced in the course of heat adaptation. The 15 increased transcriptional regulation proteins include DREB, Myb, zinc finger family proteins, MBF1, oligouridylate-binding protein, tetratricopeptide repeat-like superfamily protein, cold shock domain protein, and several RNA binding proteins (Supplementary Table S1). In addition, the phosphorylation levels of basic-leucine zipper and zinc finger CCCH domain-containing protein increased after the treatment (**Figure 8** and Supplementary Table S2). This result will springboard the investigation of heat stress-responsive transcriptional mechanisms in spinach. Post-transcriptional regulation of heat stress-responsive genes is critical for thermotolerance. It has been reported that nearly 70% of the genes were alternatively spliced in grape leaves (Jiang et al., 2017). Consistently, the present study found several heat stress-increased spliceosome proteins, such as pre-mRNA-splicing factor SYF1, serine/arginine rich splicing factor, and small nuclear ribonucleoproteins, as well as heat-increased phosphorylation of heterogeneous nuclear ribonucleoprotein (Supplementary Table S1). They will contribute to the transcriptome plasticity and proteome diversity in spinach adaptation to heat stress.

Protein synthesis was significantly induced in spinach under heat stress, because 44 out of 49 (90%) HRPs were involved in protein synthesis, including various ribosomal proteins, aminoacyl-tRNA biosynthesis enzymes, translation initiation factors, translation elongation factors, and peptide chain release factors (Supplementary Table S1). In addition, heat-induced protein phosphorylation played important roles during protein synthesis, which was evidenced by the enhanced phosphorylation levels of 60S ribosomal protein L5-2, translation initiation factor and eukaryotic peptide chain release factor (Supplementary Table S1). HSPs and other chaperones are necessary for protecting proteins from improper folding, denaturation, and aggregation under heat stress (Johnová etal., 2016). In this study, 56 of 69 (81%) HRPs involved in protein folding and processing dramatically increased in spinach under heat stress (Supplementary Table S1). Among them, 27 HSPs including HSP60/CPN60, HSP70, HSP90, HSP100/ClpB, and small HSPs, together with HSP90 activator and most of the co-chaperones (i.e., DnaJ, GrpE, CPN10, and HSP70/HSP90 organizing protein), were all heat stress-increased (Supplementary Table S1). This result is consistent with previous findings that most of the HSPs genes were dramatically up-regulated in spinach leaves under heat stress (Yan et al., 2016). Similarly, a large group of proteomic studies have reported that heat stress increased the levels of CPN60, HSP70, HSP90, small HSPs, and DnaJ in the leaves of rice (Lee et al., 2007; Han et al., 2009), soybean (Ahsan et al., 2010), *P*. *oleracea* (Yang et al., 2012), and *C*. *spinarum* (Zhang et al., 2010). Apart from HSPs, four protein disulfide isomerases and five out of 13 peptidyl-prolyl cis-trans isomerases for proper protein folding were increased in spinach by the heat stress (Supplementary Table S1). Besides these, several other chaperones, such as UDP-glucose:glycoprotein glucosyltransferase involved in glycoprotein folding in ER quality control system (Dejgaard et al., 2004), T-complex protein 1 possessing similar function with CPN60 (Gupta, 1995), chaperonin-like RbcX protein involved in assembly of RuBisCO (Saschenbrecker et al., 2007), tubulin-specific chaperone A, and PEX19 involved in importing receptor for newly synthesized class I peroxisomal membrane proteins (Fang et al., 2004), were heat stress-increased. Clearly, multiple molecular chaperones were employed in spinach for dealing with the heat stress condition. Moreover, 46 out of 72 (64%) HRPs for protein degradation were increased by the heat stress, among which 33 proteins were involved in the ubiquitin-proteasome pathway (Supplementary Table S1). Activation of the protein degradation pathway may be needed to prevent accumulation of non-functional and potentially toxic proteins in spinach under heat stress.

### Heat Stress Induces Diverse Primary and Secondary Metabolisms

In spinach, the abundance levels of enzymes involved in carbohydrate and energy metabolism increased under heat stress. For example, 5 out of 8, and 35 out of 48 HRPs were involved in glycolysis and sugar metabolism, respectively. In addition, four proteins participated in pentose phosphate pathway increased in response to heat stress (Supplementary Table S1). Furthermore, 29 out of 38 (76%) of HRPs involved in amino acid metabolism also significantly increased under heat stress (Supplementary Table S1). These changes in primary metabolism are important for osmotic homeostasis, active protein synthesis, and secondary metabolism in spinach leaves under heat stress. For example, heat-induced contents of proline and soluble sugars would be critical for osmotic homeostasis. However, the abundances of two key enzymes (i.e., choline monooxygenase and betaine aldehyde dehydrogenase) for glycine betaine biosynthesis (Yang et al., 2005; Shirasawa et al., 2006) were not changed in spinach leaves, implying that the contents of glycine betaine were not increased in spinach during long term heat response.

In addition, fatty acid metabolism increased in heat stress-treated spinach leaves as shown by the increases of acyl carrier protein (ACP), three acetyl-CoA carboxylases, and 3R-hydroxymyristoyl ACP dehydrase isoform 1 involved in saturated fatty acid biosynthesis (Supplementary Table S1). In addition, three polyunsaturated fatty acid biosynthesis-related enzymes including acyl-ACP desaturase, acyl-CoA oxidase, and acyl-CoA synthetase 5 (Supplementary Table S1) also increased, suggesting the changes in the proportion of lipid composition in spinach leaves are required for acclimation to the heat stress. It has been reported that saturation of thylakoid membrane lipids increased the thermal stability of the membranes (Thomas et al., 1986). Plastid fatty acid desaturase mutants with reduced levels of lipid unsaturation were shown to be heat-tolerant (Kunst et al., 1989). Furthermore, 16 out of 17 proteins involved in nucleotide metabolism increased in spinach leaves by heat stress (Supplementary Table S1). Purine and pyrimidine nucleotides are building blocks for nucleic acid synthesis, energy source, as well as precursors for the synthesis of primary and secondary products (Stasolla et al., 2003). Therefore, the activated nucleotide metabolism in heat-stressed spinach plays important roles during stress response.

Secondary metabolisms were induced by the heat stress in spinach. Vitamin metabolism was altered in spinach leaves, as shown by the increases of five out of eight HRPs in vitamin metabolism under heat stress (Supplementary Table S1). Among them, three proteins (cysteine desulfurase, thiamine biosynthesis protein ThiC, and thiamine thiazole synthase) are involved in vitamin B_1_ biosynthesis. Vitamin B_1_ plays critical roles in carbohydrate catabolism, NADPH and ATP synthesis, and nucleic acid formation (Rapala-Kozik et al., 2012). It has been reported that vitamin B_1_ biosynthesis was activated during plant adaptation to heat stress (Ferreira et al., 2006). In addition, heat stress-responsive riboflavin synthase, pyridoxal 5′-phosphate synthase subunit PDX1, and tyrosine aminotransferase 2 suggest that the biosynthesis of vitamin B_2_ (riboflavin)_,_ vitamin B_6_ and vitamin E (tocopherol) was induced in spinach, and these metabolites function as antioxidants in response to oxidative stress (Denslow et al., 2005; Asensi-Fabado and Munné-Bosch, 2010; Mahmood et al., 2012). Moreover, isoprenoid biosynthesis was enhanced in spinach under heat stress. This result is consistent with a previous finding that volatile isoprenoids can protect photosynthesis under heat and oxidative stresses (Vickers et al., 2009).

## Conclusion

Most spinach varieties are heat stress sensitive, while spinach Sp75 used in this study is a heat tolerant sibling inbred line. Our physiological and proteomics results indicated that Sp75 can accumulate multiple osmolytes/antioxidants and trigger diverse ROS scavenging pathways for heat stress response, although the photosynthesis was inhibited under prolonged heat stress. Importantly, the quantitative proteomics and phosphoproteomics results implied that calcium-mediated signaling, vesicular transporting, transcriptional regulation, protein processing, as well as primary and secondary metabolisms were induced for facilitating heat sensing, transduction, and adaptation. Moreover, we identified 6 candidates of heat-responsive receptors and 24 heat-responsive endomembrane trafficking-related proteins. Their abundance patterns implied that signal peptide recognition, vesicle formation, exocyst-mediated secretory, vesicle fusion, clathrin-mediated endocytosis, endosomal sorting and recycling, as well as endosomal-mediated degradation were active in plant heat response. Importantly, 7 proteins involved in Ran GTPase-mediated nuclear trafficking, as well as 15 heat-increased transcriptional regulation-related proteins were proposed to be novel heat-responsive factors in spinach (Supplementary Table S1). All these results provide novel clues toward discovering new heat perception molecules and nodes of fine-tuned heat adaptation networks. Additional studies of molecular genetic screens and protein structure-function analysis are still needed in the future.

## Author Contributions

SD conceived and designed the experiments. QZ, WC, JB, CX, JM, XC, XW, YL, and QW performed the experiments. ZZ and YS contributed analysis tools. QZ, HX, JC, and SG analyzed the data. QZ wrote the manuscript with suggestions by SD, ZZ, and SC. All authors have read and approved the final manuscript.

## Conflict of Interest Statement

The authors declare that the research was conducted in the absence of any commercial or financial relationships that could be construed as a potential conflict of interest.

## Footnotes

1 http://www.spinachbase.org/cgi-bin/spinach/index.cgi

2 http://www.ncbi.nlm.nih.gov/

3 http://www.ncbi.nlm.nih.gov/BLAST/

4 http://www.kegg.jp/kegg/

5 http://www.ebi.uniprot.org/

6 http://geneontology.org

7 http://bonsai.hgc.jp/~mdehoon/software/cluster/software.htm

8 http://swissmodel.expasy.org/

9 http://www.ebi.ac.uk/interpro/

## Abbreviations

ABC: ATP-binding cassette
ACP: acyl carrier protein
APX: ascorbate peroxidase
ARF: ADP-ribosylation factor
AsA: ascorbate
CaM: calmodulin
CAT: catalase
CBP: calcium-binding EF hand family protein
CDPK3: calcium-dependent protein kinase 3
Ci: intercellular CO_2_ concentration
DHA: dehydroascorbate
DHAR: dehydroascorbate reductase
DREB: dehydration-responsive element-binding
ER: endoplasmic reticulum
ESCRT: endosomal sorting complex required for transport
GAP: GTPase-activating protein
GOX: glycolate oxidase
GPX: glutathione peroxidase
GR: glutathione reductase
Grx: glutaredoxin
Gs: stomatal conductance
GSH: glutathione
GSSG: oxidized glutathione
GST: glutathione S-transferase
HHT: hour of heat treatment
HRP: heat stress-responsive protein
HSF: heat shock transcription factor
HSP: heat shock protein
MAPK: mitogen-activated protein kinase
MBF1: multiprotein bridging factor 1
MDA: malondialdehyde
MDHAR: monodehydroascorbate reductase
OEC: oxygen evolving complex
PLC: phospholipase C
PLD: phospholipase D
PM: plasma membrane
Pn: net photosynthetic rate
POD: peroxidase
PP2C: protein phosphatase 2C
Prx: peroxiredoxin
PSII: photosystem II
RCA: ribulose bisphosphate carboxylase/oxygenase activase
REL: relative electrolyte leakage
ROS: reactive oxygen species
RuBisCO: ribulose bisphosphate carboxylase/oxygenase
RWC: relative water content
SAR: secretion-associated and ras-superfamily-related
SOD: superoxide dismutase
Tr: transpiration rate
Trx: thioredoxin
