# Supplementary material for: Proteomics and Phosphoproteomics of Heat Stress-Responsive Mechanisms in Spinach
Source: Front Plant Sci. 2018 Jun 26;9:800. doi: 10.3389/fpls.2018.00800 (PMC6029058; doi:10.3389/fpls.2018.00800)
Supplement: Supplementary file 4 [file Image_1.PDF]

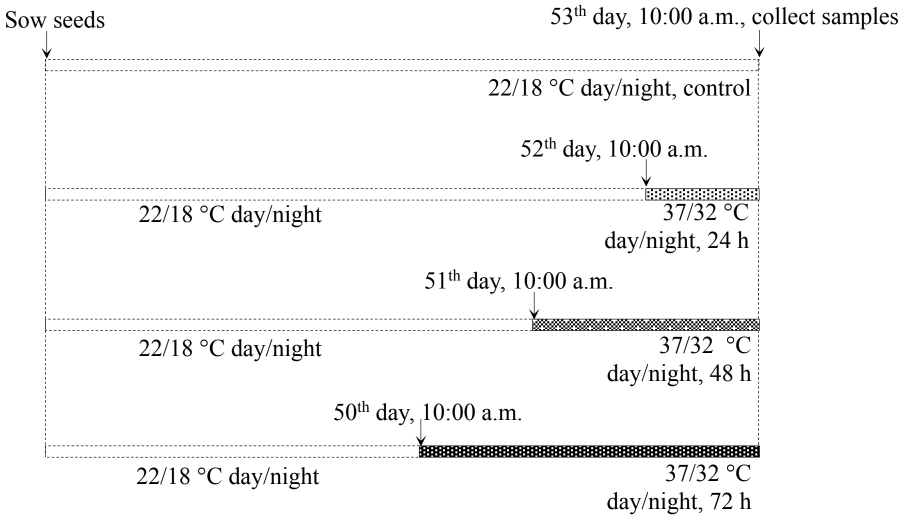

Supplemental Figure S1. Workflow of heat treatments and sample collection. A sibling inbred line of spinach (*Spinacia oleracea* L.), Sp75, was grown in a growth chamber with a temperature regime of 22/18 °C, 10/14 h day/night cycle, and a relative humidity of 60%. Plants were watered daily to avoid the occurrence of water deficit. When performing heat treatments, plants of the treatment groups were moved to another growth chamber with the same growth condition as the control, except for temperature (37/32°C day/night), and watered daily on a regular schedule as well. The 72 h of heat treatment was started at 10:00 a.m. on the 50th day after seeds were sowed, and 48 h and 24 h of heat treatments were conducted at 10:00 a.m. on the 51th day and 52th day, respectively. After heat treatments, fully expanded true leaves were collected, morphological changes were recorded, and photosynthetic characteristics were measured for both control and heat-treated plants at 10:00 a.m. on the 53th day to avoid the differences caused by plant growth, development, and circadian rhythms.
