# Supplementary material for: Proteomics and Phosphoproteomics of Heat Stress-Responsive Mechanisms in Spinach
Source: Front Plant Sci. 2018 Jun 26;9:800. doi: 10.3389/fpls.2018.00800 (PMC6029058; doi:10.3389/fpls.2018.00800)
Supplement: Supplementary file 5 [file Image_2.PDF]

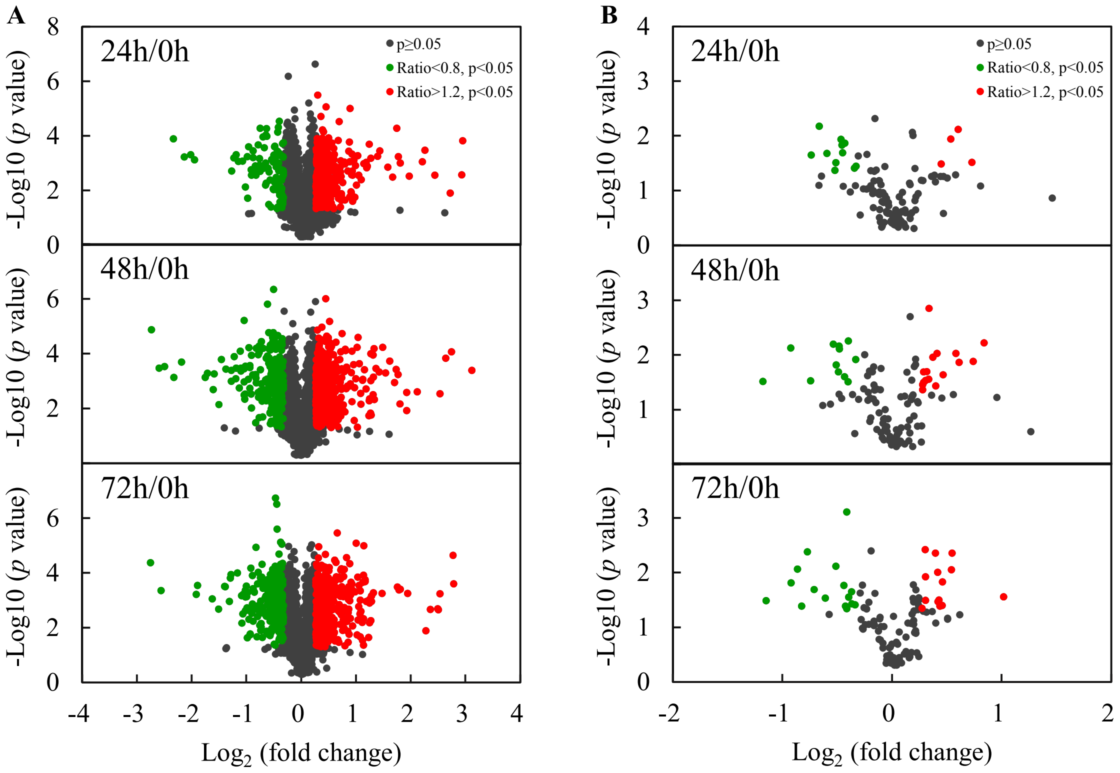

Supplementary Figure S2 Volcano plots of heat-responsive proteins and phosphopeptides. Ratios were calculated from heat-treated samples (37/32 °C day/night treated for 24 h, 48 h, and 72 h) compared with control, respectively. Negative log10 *p* values were plotted on the *y* axis with log2 normalized abundance fold change of proteins/phosphopeptides on the *x* axis. Significant differential abundances of proteins/phosphopeptides levels with *p* value threshold values α = 0.05 were present in areas exceeding the 0.8 and 1.2 of fold change threshold values. Red and green dots indicate heat-increased and decreased protein abundances/phosphorylation levels in heat-treated plants relative to control. (A) Volcano plot for heat-responsive proteins; (B) Volcano plot for heat-responsive phosphopeptides.
